# Supplementary material for: The Economic Burden of Severe Acute Malnutrition with Complications: A Cost Analysis for Inpatient Children Aged 6 to 59 Months in Northern Senegal
Source: Nutrients. 2024 Jul 10;16(14):2192. doi: 10.3390/nu16142192 (PMC11279731; doi:10.3390/nu16142192)
Supplement: Supplementary file 1 [file nutrients-16-02192-s001.zip › nutrients-3062753-supplementary.pdf]

Supplementary Materials

The Economic Burden of Severe Acute Malnutrition with Complications: A Cost Analysis for Inpatient Children Aged 6 to 59 Months in Northern Senegal

Bibata Wassonguema [1,2,*], Dieynaba S. N'Diaye [1], Morgane Michel [2,3], Laure Ngabirano [1], Severine Frison [1], Matar Ba [4], Françoise Siroma [4], Antonio V. Brizuela [5], Martine Audibert [6] and Karine Chevreul [2,3]

[1] Research Unit, Expertise & Advocacy Department, Action Contre la Faim (ACF), 93100 Montreuil, France
[2] ECEVE, UMR 1123, Université Paris Cité, Inserm, 75010 Paris, France
[3] Unité D'épidémiologique Clinique, Hôpital Robert Debré, Assistance Publique-Hôpitaux de Paris, 75019 Paris, France
[4] Action Contre la Faim, Dakar 29621, Senegal
[5] Action against Hunger, 28002 Madrid, Spain
[6] Centre d'Études et de Recherches sur le Développement International (CERDI) CNRS-IRD-UCA, 63000 Clermont-Ferrand, France
* Correspondence: bwassonguema@actioncontrelafaim.org

Table of contents

METHODS 3

Study sites 3

Materials (multiple use items) cost estimation 3

RESULTS 4

Patients' characteristics presented by status of the hospitalization facility 4

Sensitivity analysis, scenario analysis 5

Influence of patient's sociodemographic and clinical characteristics on total costs: Model robustness checking 7

Table S1: Materials useful times used in the base case analysis and in sensitivity analysis. 4
Table S2: Characteristics of children aged 6 to 59 months admitted for complicated severe acute in northern Senegal from January to December 2020, presented by status of the hospitalization facility (n=140) 4
Table S3: Worst and best cases scenario analysis of cost of inpatient stay to treat complicated severe acute malnutrition in children aged 6 to 59 months in Northern Senegal in 2020 7

METHODS

Study sites

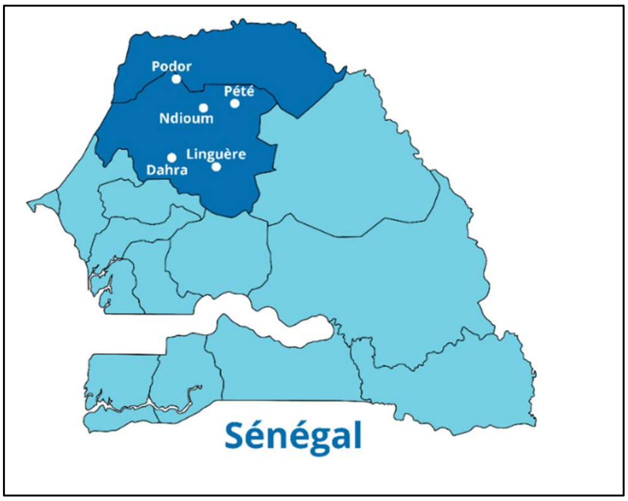

Figure S1: Study sites identified on Senegal map

Materials (multiple use items) cost estimation

The cost of items which were used for multiple patients was calculated based on the annual depreciation (Drummond et al., 2015; Johns et al., 2003) attributable to each patient. For each CREN, the annual depreciation value of each material was calculated by dividing the material cost by its useful life *(Table S1)*.

$$Material\ annual\ depreciation\ value = \frac{Material\ cost(Material\ cost - salvage\ value)}{Material\ useful\ time\ (years)}$$

We assumed the residual value (salvage value) which is the value of the asset at the end of its useful life was null. We then applied this formula:

$$Material\ annual\ depreciation\ value = \frac{Material\ cost}{Material\ useful\ time\ (years)}$$

With $Material\ cost = purshase\ cost + \ financial\ and\ operaional\ fees$

The financial and operational fees correspond to clearance taxes, insurance, custom, handling, processing and storage. For the base case analysis, these charges where considered equivalent to 50% of the purchase cost.

The annual depreciation value of all the materials were than summed reported to the number of admissions in one year to get the depreciation value attributed to each patient.

The items considered were materials commonly used for anthropometric measurements (weighting scale, height-measuring system) and for medical examination (thermometer, stethoscope, and oximeter). The useful lives were estimated from expert (experienced in using these items) opinion.

Table S1: Materials useful times used in the base case analysis and in sensitivity analysis.

Material	Useful time (in years), base case analysis	Useful time (in years), best-case scenario. Corresponds to expert lowest value declared	Useful time (in years), worst case scenario Corresponds to expert highest value declared
Weighting scale	4	3	5
Height-measuring system	5,5	4	7
Thermometer	0.375	0.25	0.5
Stethoscope	1.5	2	3
Oximeter	2	1	3

RESULTS

Patients' characteristics presented by status of the hospitalization facility

Table S2: Characteristics of children aged 6 to 59 months admitted for complicated severe acute in northern Senegal from January to December 2020, presented by status of the hospitalization facility (n=140)

**Health facility characteristics**	**Hospital based CREN (N=89)**	**Health centre based CREN (N=51)**
**Socio-demographic characteristics**		
Sex, n (%)		
Male	54 (60.7 %)	21 (41.2%)
Female	35 (39.3%)	30 (58.8%)
Age in months, mean (sd)	17.6 (9.3)	18.8 (9.7)
Residence distance from the CREN, n (%)		
≤10 km	24 (27.0%)	19 (37.3%)
11-50 km	44 (49.4%)	21 (41.2%)
>50 km	21 (23.6 %)	11 (21.6%)
**Anthropometric measurements**		
Weight at admission, in kg, mean (sd)	6.9 (1.9)	6.8 (1.4)
Height at admission, in cm, mean (sd)	75.0 (9.4)	75.2 (8.7)
Severe acute malnutrition severity, n (%)		
Severely wasted (-4 < z-score ≤ -3)	25 (28.1%)	19 (37.5%)

Very severely wasted (z-score ≤ -4)	64 (71.9%)	32 (62.6%)
**Characteristics of the stay**		
Admission type, n (%)		
New admission[†]	89 (100%)	48 (94.1%)
Relapse[‡]	0 (0%)	3 (5.9%)
Admission mode, n (%)		
Referral from UREN[§]	72 (80.9%)	45 (88.2%)
Spontaneous screening[¶]	17 (19.1%)	6 (11.8%)
Type of discharge from the CREN, n (%)		
Successfully treated[#]	72 (80.9%)	46 (90.2%)
Dropped out[‖]	9 (10.1%)	2 (3.9%)
Medical referral[††]	0 (0.0%)	1 (2.0%)
Dead	8 (9%)	2 (3.9%)
Length of stay, in days, mean (sd)	5.3 (3.2)	5.3 (3.3)
**Complications motivating the admission *(not mutually exclusive)*, n (%)**		
Diarrhoea	48 (53.9%)	30 (58.8%)
Dehydration	27 (30.3%)	14 (27.5%)
Acute respiratory infection	15 (16.9%)	12 (23.5%)
Anaemia	31 (22.1%)	0 (0%)
Anorexia	11 (12.4 %)	3 (5.9%)
Oedema	4 (7.8%)	1 (1.1%)
Other[‡‡]	9 (10.11%)	3 (5.9%)
Number of complications, n (%)		
1	45 (50.6 %)	32 (62.8%)
2	31 (34.8 %)	16 (31.4%)
3-4	13 (14.6%)	3 (5.9%)
Number of complications, mean (sd)	1.7 (0.8)	1.5 (0.7)

[†]Complications treated and child referred back to UREN to continue severe acute malnutrition outpatient treatment
[‡]Decision to leave the CREN against medical advice
[§]Referral to another inpatient facility
[#]Other complications include hyperthermia, oral candidiasis, sickle cell disease, psychomotor disability, trisomy and asthma

Abbreviations:
CREN = Centre for Rehabilitation and Nutritional Education, SAM inpatient treatment centre in Senegal
SD = Standard deviation
UREN: Unit for Rehabilitation and Nutritional Education, SAM outpatient treatment centre in Senegal
km=kilometre

Sensitivity analysis, scenario analysis

Table S3 presents the results of worst and best cases scenario analysis we performed as part of sensitivity analysis, to assess uncertainty surrounding the cost estimation. Results are presented as an uncertainty range, with the lower bound corresponding to the estimated cost with the best case scenarii made on the study parameters, and the lower bound to the estimated cost with the worst case scenarii.

Table S3: Worst and best cases scenario analysis of cost of inpatient stay to treat complicated severe acute malnutrition in children aged 6 to 59 months in Northern Senegal in 2020

	BASECASE SCENARIO Mean cost (sd) (in 2020 US international $†)	SCENARIO ANALYSIS Uncertainty range‡ (in 2020 US international $†)
**Direct medical costs**	**320.5 (154.3)**	**[182.9- 488.2]**
**Borne by the health system**	**201.7 (103.7)**	**[86.0 – 344.9]**
Personnel	142.7 (84.4)	[45.9 - 261.4]
Drugs	4.6 (6.8)	[4.1 – 5.1]
Therapeutic foods	35.5 (21.9)	[19.7 - 57.2]
Hospital bed	2.2 (4.2)	[2.0 – 2.4]
Material and consumables	16.5 (4.0)	[14.3 - 18.9]
**Borne by households**	**118.9 (82.5)**	**[96.9 – 143.3]**
Drugs	26.7 (24.5)	[24.0 – 29.4]
Medical tests	49.3 (34.6)	[44.4 - 54.2]
Hospital bed	42.9 (36.7)	[28.5 – 59.7]
**Direct non-medical costs**	**76.7 (35.7)**	**[58.2 - 164.6]**
**Borne by households**	**76.7 (35.7)**	**[58.2 - 164.6]**
Caregiver(s)§ meals	48.4 (29.3)	[32.7 - 133.3]
Transport	20.0 (17.4)	[18 - 22]
Hygiene kit	8.3 (0.0)	[7.4 - 9.3]
**Total direct costs**	**393.2 (30.0)**	**[241.0 – 651.8]**
**Indirect costs (caregiver productivity loss)**	**34.7 (21.0)**	**[19.5 - 108.4]**
**Total Costs**	**431.9 (203.9)**	**[260.6 – 761.2]**
**Paid by the health system**	**201.6 (103.7)**	**[86.0- 344.9]**
**Paid by the households (out of pocket)**	**195.6 (103.6)**	**[155.1 – 307.9]**
**Caregiver(s) productivity loss**	**34.7 (21.0)**	**[19.5 - 108.4]**

† 2020 US international dollar using purchasing power parity (PPP) exchange rate of CFA *vis* dollar: 1$= 240.317 XOF.
‡ The lower bound corresponds to the estimated cost for the best-case scenario in the sensitivity analyses, and the upper bound

Influence of patient's sociodemographic and clinical characteristics on total costs: Model robustness checking

Regression model fit checking - model check for linearity

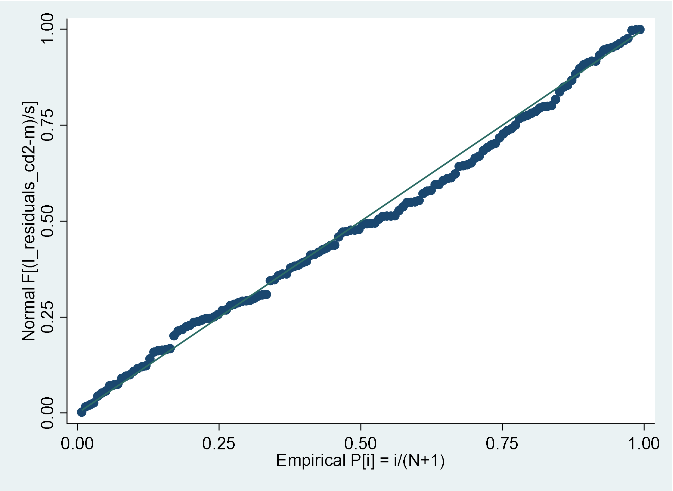

Figure S2: Regression model fit checking - model check for linearity, points falls on X=Y line

Regression model fit checking for normality of residuals

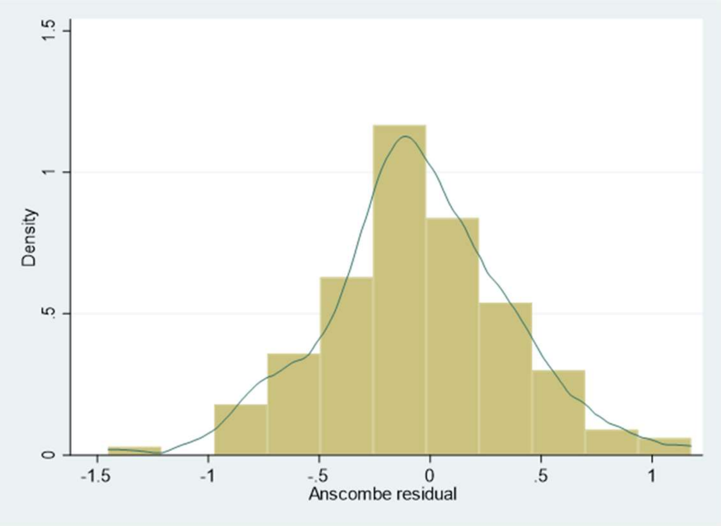

Shapiro-Wilk W test for normal data

Variable	Obs	W	V	z	Prob>z
l_residua~d2	140	0.98715	1.410	0.776	0.21889

Figure S3 : Regression model fit checking for normality of residuals, the residuals distribution looks normal and the H0 hypothesis of Shapiro-Wilk test of normality was not rejected (pvalue>0.05).

REFERENCES

Drummond, M. F., Sculpher, M. J., Claxton, K., Stoddart, G. L., & Torrance, G. W. (2015). *Methods for the Economic Evaluation of Health Care Programmes*. Oxford University Press.

Johns, B., Baltussen, R., & Hutubessy, R. (2003). Programme costs in the economic evaluation of health interventions. *Cost Effectiveness and Resource Allocation : C/E*, *1*, 1. https://doi.org/10.1186/1478-7547-1-1
